# Quantitative assessment of active charcot neuro-osteoarthropathy in diabetes using ^18^F-sodium fluoride PET/CT

**DOI:** 10.3389/fendo.2026.1930281

**Published:** 2026-09-25

**Authors:** Jayaditya Ghosh, Ashu Rastogi, Avanthiga Subrhamanian, Harmandeep Singh, Anindita Sinha, Raghuraman Soundararajan, Pinaki Dutta, Sanjay Kumar Bhadada

**Affiliations:** 1 Department of Endocrinology, Post Graduate Institute of Medical Education and Research, Chandigarh, India; 2 Department of Nuclear Medicine, Post Graduate Institute of Medical Education and Research, Chandigarh, India; 3 Department of Radiodiagnosis, Post Graduate Institute of Medical Education and Research, Chandigarh, India

**Keywords:** 18F-sodium fluoride positron emission tomography- computed tomography, charcot neuro-osteoarthropathy, MRI, recurrence, remission

## Abstract

**Background:**

No single imaging modality currently provides quantitative assessment of disease activity in active charcot neuro-osteoarthropathy (CNO) of the foot, either at diagnosis or during follow-up after clinical remission. This study evaluates the role of ^18^F-sodium fluoride positron emission tomography–computed tomography (^18^F-NaF PET/CT) in active CNO.

**Methods:**

In this prospective study, 80 patients with diabetes presenting with asymmetric foot swelling were screened and assessed using an infrared thermometer. 39 symptomatic patients were included (33 active CNO and 6 non-CNO) and underwent magnetic resonance imaging (MRI) and ^18^F-NaF PET/CT. 20 additional participants (10 diabetic neuropathy and 10 healthy controls) underwent PET/CT for assessing the uptake in diabetic patients and normal individuals. Standardized uptake value maximum (SUVmax) on ^18^F-Sodium Fluoride PET-CT was measured at the region of interest (ROI) of affected foot at baseline, clinical remission, and three-monthly post-remission. Change in SUVmax from baseline to clinical remission was compared between patients with sustained remission and those with relapse. Exploratory univariate Cox regression analysis was performed to assess associations between SUVmax changes and recurrence.

**Results:**

Median SUVmax (baseline) was 55.1 in active CNO versus 7.5 in non-CNO (p<0.001). An SUVmax value of ≥10.3 yielded 100% sensitivity (95% CI 89.4–100%) and 92.3% specificity (95% CI 74.9–99.1%) against all controls (AUC 0.988, 95% CI 0.965–1.000); specificity was 66.7% against symptomatic non-CNO patients. 29 participants achieved clinical remission, and SUVmax decreased from 58.3 to 19.6 (61.9% reduction; p<0.001). 4 patients with active CNO relapse showed significantly lower SUVmax reduction than those with sustained remission (28.4% vs 64.7%, p=0.006). In exploratory analysis, greater reduction in SUVmax between baseline and clinical remission was associated with lower recurrence risk (HR 0.970 per 1% greater reduction, 95% CI 0.941–0.999). With only four events, this finding is hypothesis-generating.

**Conclusion:**

^18^F-NaF PET/CT shows promise as a quantitative marker of bone remodelling in active CNO in patients with diabetes. These findings are exploratory and require external validation in large cohorts.

## Introduction

Charcot neuro-osteoarthropathy (CNO) is a severe complication primarily affecting people with diabetes. Reported prevalence ranges from approximately 0.07% in the general diabetic population to as high as 13% in selected high-risk populations, particularly those with peripheral neuropathy, reflecting differences in study populations and case ascertainment (1). Unilateral active CNO of foot presents with asymmetric foot swelling with associated signs of inflammation, including redness and warmth. But the diagnosis of active CNO is often delayed or missed because of common masqueraders, highlighting the limitations of clinical examination (2). Current recommendations suggest infrared thermography for monitoring CNO remission, with remission defined as a temperature difference of less than 2 °C (3). However, despite clinical remission, an underlying destructive process continues contributing to a higher risk of recurrence and deformities (12–33%) (4, 5). Therefore, X-ray and magnetic resonance imaging (MRI) are commonly used to confirm the diagnosis and monitor active CNO. However, the accuracy of X-ray is limited for the early diagnosis of CNO, and MRI does not aid in quantitative monitoring for remission or assessing recurrence of active CNO.

Nuclear imaging techniques, including skeletal scintigraphy and single-photon emission computed tomography (SPECT), have varying sensitivity for the CNO diagnosis (6). Looking at the pathophysiology of CNO, it is characterized by accelerated bone remodeling, resulting in progressive joint and bone destruction. ^18^F-Sodium Fluoride (^18^F-NaF) exhibits a remarkably high affinity for bone, particularly in regions of active remodeling, and clears quickly from soft tissues, making it an excellent radiotracer for skeletal pathologies (7). Although MRI remains the preferred imaging modality for evaluating active CNO (8), objective quantification of disease activity and treatment response remains challenging. The published experience with ^18^F-NaF PET/CT in active CNO is extremely limited, with only a single case series involving three patients reported to date (9). In contrast, ^18^F-NaF PET/CT has been more extensively investigated as a quantitative marker of bone remodeling in skeletal malignancies, particularly for the detection and assessment of bone metastases (10). Although the limited published experience suggests that ^18^F-NaF PET/CT can detect increased bone remodeling and also may be in active CNO, but its diagnostic accuracy, quantitative role in monitoring disease activity, and potential utility for assessing recurrence have not been systematically evaluated. The primary objective of this study was to evaluate the role of ^18^F-NaF PET/CT in the diagnosis of active CNO and in monitoring disease activity during treatment. A secondary, exploratory objective was to assess whether changes in quantitative PET parameters, including SUVmax, were associated with disease recurrence following remission.

## Materials and methods

This is a prospective, observational study conducted in the Department of Endocrinology, Postgraduate Institute of Medical Education and Research, Chandigarh, India from October 2022 to December 2024. The diagnosis of active CNO was established by the treating clinician according to criteria prespecified in the study protocol: a clinically warm, swollen foot with an inter-foot temperature difference >2 °C in a patient with peripheral neuropathy, supported by MRI evidence of bone marrow oedema in a characteristic distribution without sinus tract or ulceration, and confirmed by the subsequent clinical course during offloading. MRI studies were interpreted by two radiologists blinded to the ^18^F-NaF PET/CT findings, and PET/CT studies were interpreted by a nuclear medicine physician blinded to the MRI findings. ^18^F-NaF PET/CT results did not contribute to the initial diagnosis of active CNO and were not available to the clinician establishing the diagnosis.

Patients were excluded if they had active foot ulcers, had contraindications to MRI or on antiresorptive agents (denosumab or bisphosphonates within the past year) and no participant was receiving other medication known to affect bone turnover on extensive historical assessment. Comprehensive history regarding the diabetes treatment was also taken.

The study protocol was approved by the Institute’s Ethical Committee (IEC-INT/2022/DM-764), and written informed consent was obtained from all the participants.

All the patients meeting the inclusion criteria were evaluated with a thorough history regarding the onset of foot swelling. A comprehensive foot examination was performed, including neuropathy and vasculopathy assessment. Peripheral neuropathy was defined as loss of protective sensation assessed with Semmes-Weinstein 10-g monofilament and/or the vibration perception threshold >25mV. Biochemical tests, including calcium, phosphate, albumin, alkaline phosphatase, parathyroid hormone, and 25-hydroxy-vitamin D, fasting blood glucose, postprandial blood glucose, HbA1c, lipid profile, beta C-terminal cross-linked telopeptide of type I collagen (βCTX), and procollagen type I N-terminal propeptide (P1NP), were performed.

Dermal thermometry was performed with an infrared thermometer (FLIR Systems Inc, Orlando, USA) with a pixel resolution of 4800 (80*60), thermal sensitivity of 0.15 C, a detection range of -20 °C to 250 °C, and a coefficient of variation of 2%, from the standardized height of knee length in a centralized temperature-regulated room of 24 °C. An average of three temperature readings was obtained at the Region of Interest (ROI) of the foot.

### MRI

Weight-bearing x-ray (Anteroposterior and lateral) and MRI of the bilateral feet were performed. MRI sequences included T1-weighted, T2-weighted, diffusion-weighted imaging (DWI), and apparent diffusion coefficient (ADC) were obtained with a 3T scanner (Siemens Magnetom Verio). T2-weighted hyperintensity in the bones indicated bone marrow edema, and its extent was recorded. Active CNO was diagnosed based on the presence of diffuse T2-hyperintense areas in the bone and surrounding muscle (8) without any associated sinus or tract. Bone-marrow edema detected on T2-weighted imaging was distinguished from osteomyelitis using DWI and ADC mapping. Patients with active foot ulceration or clinically evident infection were excluded, substantially reducing the prior probability of osteomyelitis, which in the diabetic foot arises predominantly by contiguous spread from an overlying ulcer. Where bone marrow oedema required further characterisation, DWI with ADC mapping was used to distinguish osteomyelitis from CNO-related oedema, applying the criterion of, in which ADC values below 1.57 × 10^-^³ mm²/s favour osteomyelitis (11). No patient in the active CNO group was diagnosed with concurrent osteomyelitis.

The diagnosis of active CNO was established according to current International working group on diabetic foot (IWGDF) guidance, which recommends diagnosis on the basis of clinical assessment of a warm, swollen foot in a person with peripheral neuropathy, supported by MRI where the diagnosis is uncertain. MRI served as the principal imaging reference and was interpreted in conjunction with the clinical presentation but the radiologists were blinded to clinical and PET scan findings (Group A and Group B). The final diagnosis of active CNO was based on the composite assessment of clinical findings and MRI features.

### ^18^F-NaF PET-CT

^18^F-NaF PET/CT was performed using a PET/CT scanner (GE Healthcare, Discovery STE16/Discovery 710). Both scanners were calibrated according to NEMA standards and cross-calibrated with the dose calibrator to ensure accuracy of SUV measurement. Patients were not required to fast before the scan, and adequate hydration was ensured. ^18^F-NaF was administered intravenously at a dose of 4–5 mCi (148–185 MBq), and regional PET/CT of the bilateral feet was acquired 45–60 minutes after injection. CT was acquired and used for attenuation correction and anatomical localization. PET images were acquired for 90 seconds per bed position and were reconstructed using an iterative algorithm (VUE Point FX, 3 iterations, 24 subsets) with SharpIR, a light z-axis filter with a 5.5 mm cutoff, and a matrix size of 192 × 192. SUV was normalized to body weight. For each scan, the region of highest tracer uptake within the affected foot was identified on the fused PET/CT images. A fixed-size three-dimensional volume of interest of 1.2 cm³ was placed at this site and SUVmax recorded, with the underlying bone identified anatomically on the co-registered CT component. On all follow-up scans, the volume of interest was placed over this same anatomically defined bone, identified using CT landmarks, so that serial measurements referred to a consistent skeletal site. All measurements were performed by a single nuclear medicine physician (HS) with 10 years of experience, blinded to the MRI findings. All serial scans in an individual patient were performed on the same system. The percentage change in SUVmax from baseline to remission was calculated [Delta SUVmax= (((baseline SUVmax- remission SUVmax)/baseline SUVmax) *100)]. In case of calculation of SUVmax ratio (SUVmax(R)) at the ROI = Affected foot SUVmax/Unaffected foot SUVmax, Whereas SUVmax(R) change (%) = [(Baseline SUVmax(R)- Remission SUVmax(R))/Baseline SUVmax(R)]​×100.

^18^F-NaF PET-CT was performed at diagnosis, clinical remission, and 3 months subsequently following remission of active CNO. We also performed ¹^8^F-NaF PET-CT scans for 10 healthy non-diabetic individuals and 10 individuals with diabetes and peripheral neuropathy for comparison with active CNO.

### Treatment

All patients diagnosed with active CNO (Group A) were treated with a non-removable, fiberglass, knee-high total contact cast (TCC). The cast was replaced every month or earlier (if needed). Infrared dermal thermometry was performed to assess the inter-foot temperature difference at each visit after the removal of the TCC. Offloading was delivered by non-removable total contact casting in all patients, minimising variation in adherence.

Remission of active CNO was defined as resolution of signs of inflammation on clinical examination and an interfeet temperature difference of less than 2 °C (documented on two occasions one month apart).

### Follow-up protocol

All participants were followed monthly after clinical remission, with an additional ^18^F-NaF PET-CT performed at three-monthly intervals to assess ongoing bone activity. “Clinical recurrence” was defined as an inter-feet temperature difference > 2 °C on two consecutive measurements one month apart following initial clinical remission. Delta SUVmax, measured from baseline to clinical remission. The three-month post-remission ^18^F-NaF PET/CT was protocol-mandated in all participants irrespective of clinical status, was undertaken for descriptive purposes, and was not included in Delta SUVmax. ^18^F-NaF PET/CT results were not disclosed to the treating clinical team at any point during the study. Decisions regarding cast duration, declaration of clinical remission, and identification of recurrence were made solely on clinical grounds and infrared dermal thermometry, according to predefined criteria as per IWGDF, without knowledge of PET findings.

Patients with sustained remission (Group A1) were compared with those who developed recurrence (Group A2) to explore factors associated with recurrence.

### Statistical analysis

The statistical analyses were performed under the supervision of an experienced biostatistician using SPSS 30.0. We conducted the Kolmogorov-Smirnov test to assess the normality of the data, and the results indicated that the data were not normally distributed. The Mann-Whitney U test was used to compare SUVmax between the two groups (with and without active CNO) and between the sustained remission and recurrence subgroups. Receiver operating characteristic (ROC) analysis was performed to evaluate the discriminatory performance of baseline SUVmax for active CNO. The diagnostic threshold was selected using a maximum-sensitivity criterion rather than the Youden index, reflecting the clinical priority of avoiding missed active CNO; the highest SUVmax value retaining 100% sensitivity was selected, with the corresponding specificity accepted as the resulting value. Areas under the curve are reported with DeLong 95% confidence intervals, and exact (Clopper–Pearson) 95% confidence intervals are reported for sensitivity and specificity.

Internal validation used 10, 000 stratified bootstrap resamples in which the complete threshold-selection procedure was repeated within each resample and the resulting threshold applied to the original cohort. Optimism was estimated as the mean difference in performance between the resample and the original data and subtracted from the apparent estimates. Leave-one-out cross-validation of the same procedure was performed as a secondary check. Bootstrap and cross-validation analyses were performed in Python 3.11 (scikit-learn, SciPy); all other analyses were performed in SPSS 30.0. As the control group comprised both symptomatic patients in whom active CNO was clinically suspected and asymptomatic comparison groups, diagnostic performance was assessed in three prespecified comparisons: against all 26 controls; against symptomatic non-CNO patients together with participants with diabetic neuropathy; and against the six symptomatic non-CNO patients alone, the last representing the clinically relevant differential diagnosis.

Comparison of baseline and follow-up data was done using the Wilcoxon signed-rank test. Given the small number of recurrence events, exploratory univariate Cox proportional hazards analyses were performed. As all recurrence events occurred at the same time point, the exact method was used for handling tied event times. The percentage change in SUVmax from baseline to clinical remission (Delta SUVmax) was designated the primary variable of interest; the corresponding change in SUVmax(R) was examined as a supportive analysis, and the two variables are not independent. No adjustment for multiple comparisons was applied. Hazard ratios with 95% confidence intervals are reported. Given four events, these analyses are reported as hypothesis-generating, with emphasis on effect estimates and confidence intervals rather than statistical significance.

## Results

The patient disposition is shown in **Figure 1**, and the baseline patient characteristics are described in **Table 1**. The median SUV max in Group A (active CNO) was 55.1 (45.6-63.4) and in Group B was 7.5 (7.13-11.25) (**Figure 2**) (P<0.001). Median SUVmax was 6.4 in diabetic neuropathy (control 1) and 2.4 healthy (control 2). ROC analysis included 33 patients with active CNO and 26 controls (6 symptomatic non-CNO patients, 10 with diabetic neuropathy, 10 healthy controls). Against all controls, baseline SUVmax discriminated active CNO with an AUC of 0.988 (95% CI 0.965–1.000). Applying the maximum-sensitivity criterion, a threshold of SUVmax 10.3 yielded sensitivity 100% (33/33; 95% CI 89.4–100%) and specificity 92.3% (24/26; 95% CI 74.9–99.1%).

**Figure 1:**
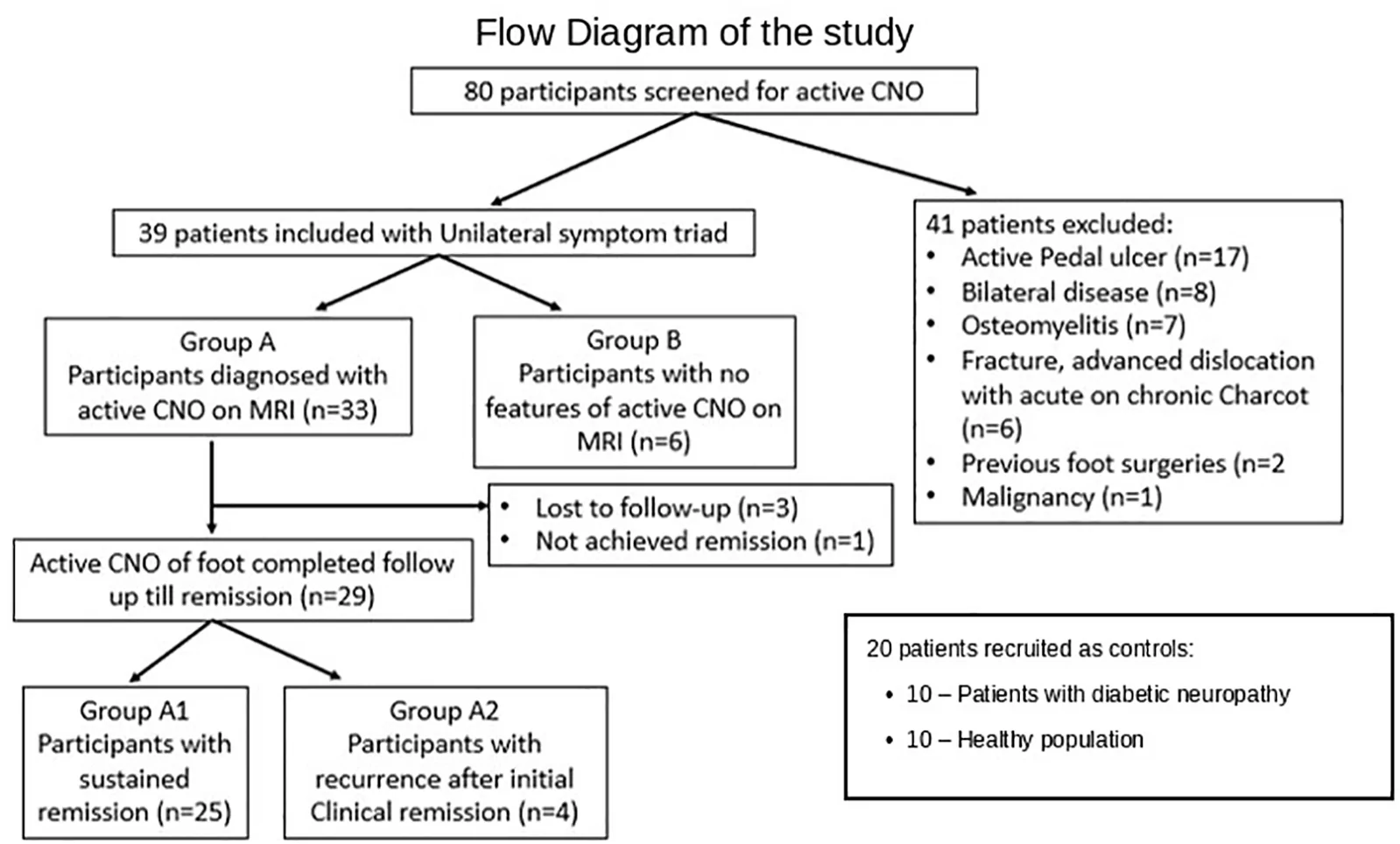
Patient disposition of the included participants.

**Table 1:** Baseline characteristics of the study population.

Baseline parameters	Median (inter-quartile range)
Age (yrs)	51 (47–59)
Sex (Male/Female)	20/19
BMI (kg/m²)	26.07 (23.38–29.41)
Duration of Diabetes (yrs)	15 (10–20)
Peripheral Arterial Disease	0/39
Diabetic Neuropathy	39/39 (VPT >25 mV: 3; VPT >40 mV: 2; VPT >50 mV: 34)
Baseline temperature difference (°C)	3.7 (3.1–5.1)
Duration of symptoms to treatment initiation (months)	5 (2–10)
Sanders–Frykberg (SF) involvement	I (forefoot) – 1 II (tarsometatarsal) – 23 III (midtarsal) – 24 IV (ankle) – 10 V (calcaneus) – 6
Eichenholtz classification	Stage 0 – 3 Stage 1 – 25 Stage 2 - 5
Previous active Charcot history	None
Hb (gm%)	12 (10.6–13.8)
Creatinine (mg/dl)	0.83 (0.67–1.25)
Estimated glomerular filtration rate (ml/min/1.73 m²)	94 (67.5–108)
Calcium (mg/dl)	9.6 (9.2–9.96)
Phosphorous (mg/dl)	3.57 (3.24–4.1)
Albumin (mg/dl)	4.34 (4.22–4.5)
ALP (U/L)	108 (94–144)
25(OH)D (ng/ml)	27.8 (19.4–42.2)
iPTH (pg/ml)	42.3 (34.5–57.1)
βCTX (pg/ml)	258 (201–527)
P1NP (ng/ml)	49.8 (35.6–77)
BMD (g/cm²) of spine	0.912 (0.819–1.005)

Values are median (interquartile range) unless stated otherwise. Demographic, clinical and biochemical parameters relate to the 39 symptomatic patients. Sanders–Frykberg involvement and Eichenholtz stage relate to the 33 patients with confirmed active CNO; patients could have more than one Sanders–Frykberg region involved, and counts therefore exceed the number of patients.

VPT, vibration perception threshold; BMI, body mass index; ALP, alkaline phosphatase; iPTH, intact parathyroid hormone; βCTX, beta C-terminal cross-linked telopeptide of type I collagen; P1NP, procollagen type I N-terminal propeptide; BMD, bone mineral density.

**Figure 2:**
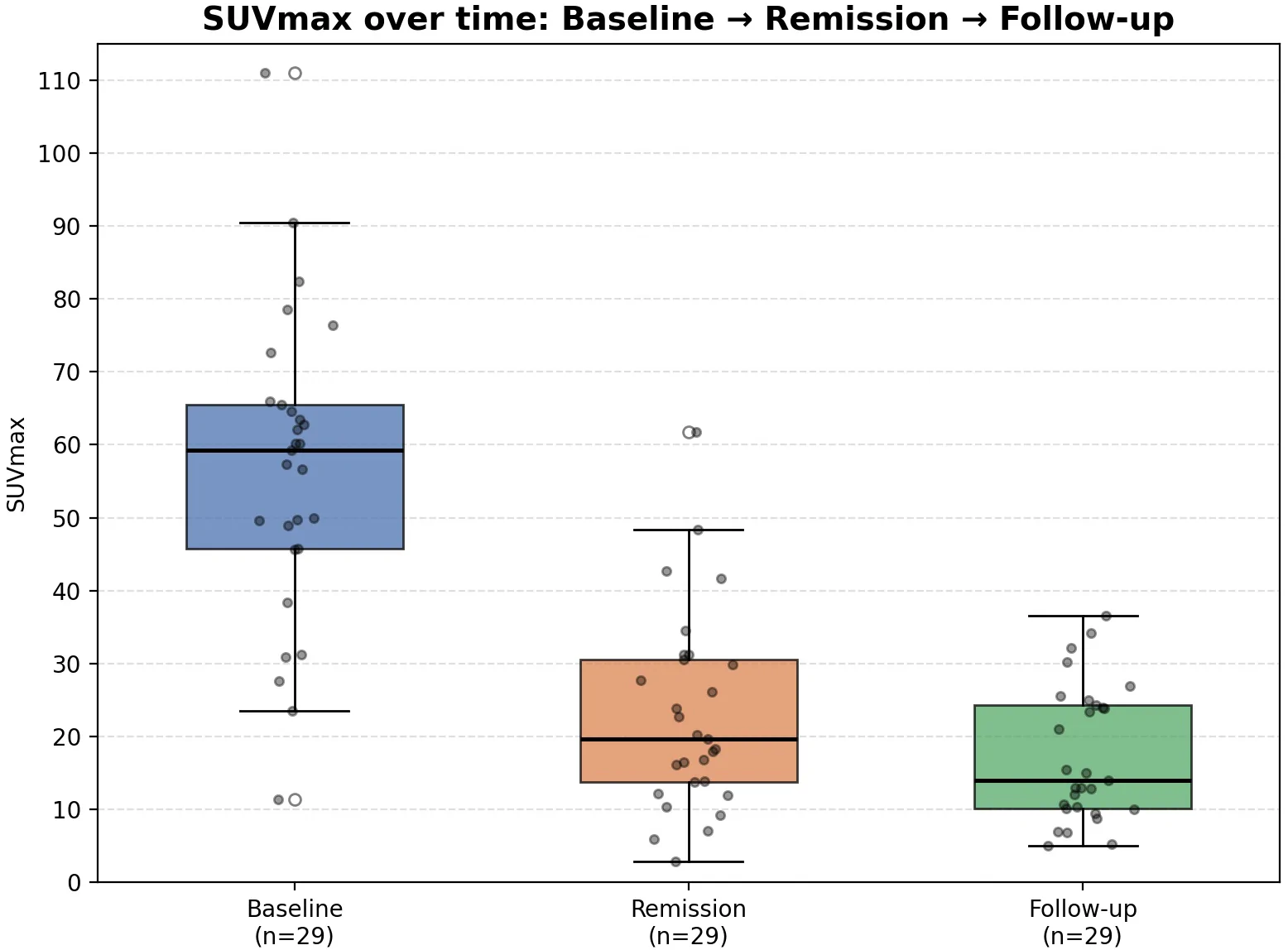
Box-Plot graph showing the trend in SUVmax Change in patients of active Charcot Neuro-osteoarthropathy of foot from baseline to remission and follow- up.

In the prespecified subgroup analyses, the AUC was 0.980 (95% CI 0.943–1.000) against symptomatic non-CNO patients and participants with diabetic neuropathy combined, with sensitivity 100% (95% CI 89.4–100%) and specificity 87.5% (95% CI 61.7–98.4%) at the 10.3 threshold. Against the six symptomatic non-CNO patients alone, the AUC was 0.947 (95% CI 0.850–1.000), with sensitivity 100% (95% CI 89.4–100%) and specificity 66.7% (95% CI 22.3–95.7%). Both false-positive results occurred within this group.

The six symptomatic non-CNO patients comprised three with cellulitis, one with soft tissue infection, one with foot trauma, and one with a stress fracture. Both false-positive results occurred in patients with underlying bone pathology (stress fracture, foot trauma), whereas all four patients with soft-tissue processes had SUVmax values below the threshold (range 5.0–7.8).

An SUVmax threshold of ≥10.3 provided high discrimination in the study cohort (AUC 0.988, 95% CI 0.965–1.000) and retained high sensitivity under internal validation (optimism-corrected sensitivity 98.9%; leave-one-out cross-validation sensitivity 97.0%). Sensitivity was not fully robust to resampling: in 35.9% of bootstrap resamples the derived threshold would have missed at least one CNO case, largely reflecting the influence of a single case with a baseline SUVmax of 11.4 against a next-lowest value of 23.5.

Among the 33 patients with active CNO, three were lost to follow-up. Of the remaining 30 patients, 29 achieved clinical remission, while one failed to achieve remission and was excluded from the remission analysis (**Figure 1**). The median time duration to achieve remission of 20 weeks (12–24). The median SUVmax decreased by 61.9% (38.63–79.86) (n=29) [baseline median SUVmax 58.3 (46.5–65.23) to 19.6 (13.7–30.5) at remission (p<0.001)].

Participants were followed up after clinical remission for a median duration of 12 months (IQR, 10-14). Four patients (13.8%) had recurrence of active CNO (3 months post-clinical remission). (**Figure 2**) The baseline data of the patients in subgroup A1 and A2 are given in **Table 2**. Overall, a decrease in Delta SUV max of 64.7% (52.01-80.47) and 28.4% (-4.07, 30.06) (p = 0.006) was observed in subgroups A1 and A2, respectively. (Representative patient data in **Figure 3**). Four recurrence events occurred among 29 patients over 321 person-months of follow-up from clinical remission (median follow-up 12 months, IQR 10–14), corresponding to a recurrence rate of 15.0 per 100 person-years. All four recurrences occurred 3 months after clinical remission. Greater reduction in SUVmax between baseline and clinical remission was associated with lower recurrence risk (HR 0.970 per 1% greater reduction, 95% CI 0.941–0.999). The change in SUVmax(R), which was highly correlated with DeltaSUVmax (Pearson r = 0.91), gave a concordant estimate (HR 0.904 per 1%, 95% CI 0.818–0.999); (**Table 3**) as these variables measure the same underlying quantity, this is presented as a supportive rather than an independent finding.

**Table 2:** Baseline characteristics comparison between the groups A1 and A2.

Parameter	Sustained remission (A1, n = 25)	Recurrence (A2, n = 4)
Age (years)	55 (48–59)	46 (44–48)
BMI (kg/m²)	29.2 (26.4–31.2)	29.3 (27.7–30.9)
Duration of diabetes (years)	15 (10–20)	5 (4–8)
Duration of symptoms (months)	5.0 (3.0–7.0)	5.5 (4.8–6.5)
Baseline inter-feet temperature difference (°C)	3.5 (2.8–4.8)	4.2 (4.1–5.0)
Baseline SUVmax	60.1 (49.6–65.9)	39.9 (29.0–51.7)
Hemoglobin (g/dL)	11.9 (10.5–13.8)	13.1 (12.2–13.8)
Creatinine (mg/dL)	0.86 (0.65–1.25)	0.79 (0.76–0.85)
Calcium (mg/dL)	9.53 (9.20–9.84)	9.70 (9.43–9.88)
Phosphorus (mg/dL)	3.56 (3.24–4.00)	3.96 (3.38–4.48)
Albumin (g/dL)	4.34 (4.22–4.43)	4.65 (4.44–4.81)
Alkaline phosphatase (U/L)	110 (99–148)	110 (100–117)
25(OH) Vitamin D (ng/mL)	23.6 (19.0–42.2)	40.5 (37.9–44.2)
Intact PTH (pg/mL)	42.3 (33.2–57.1)	39.4 (33.7–44.3)
β-CTX (pg/mL)	325 (198–527)	336 (258–480)
PINP (ng/mL)	49.8 (35.6–73.1)	43.4 (31.4–67.0)
HbA1c (%)	8.1 (7.3–8.8)	7.8 (7.2–8.7)
Total cholesterol (mg/dL)	146.7 (128.6–178.2)	132.1 (116.5–140.4)
LDL cholesterol (mg/dL)	71.0 (59.0–98.0)	48.6 (35.0–69.2)
HDL cholesterol (mg/dL)	44.0 (40.4–48.0)	37.5 (34.7–46.9)
Triglycerides (mg/dL)	163.1 (118.0–215.7)	158.6 (137.6–185.4)

**Figure 3:**
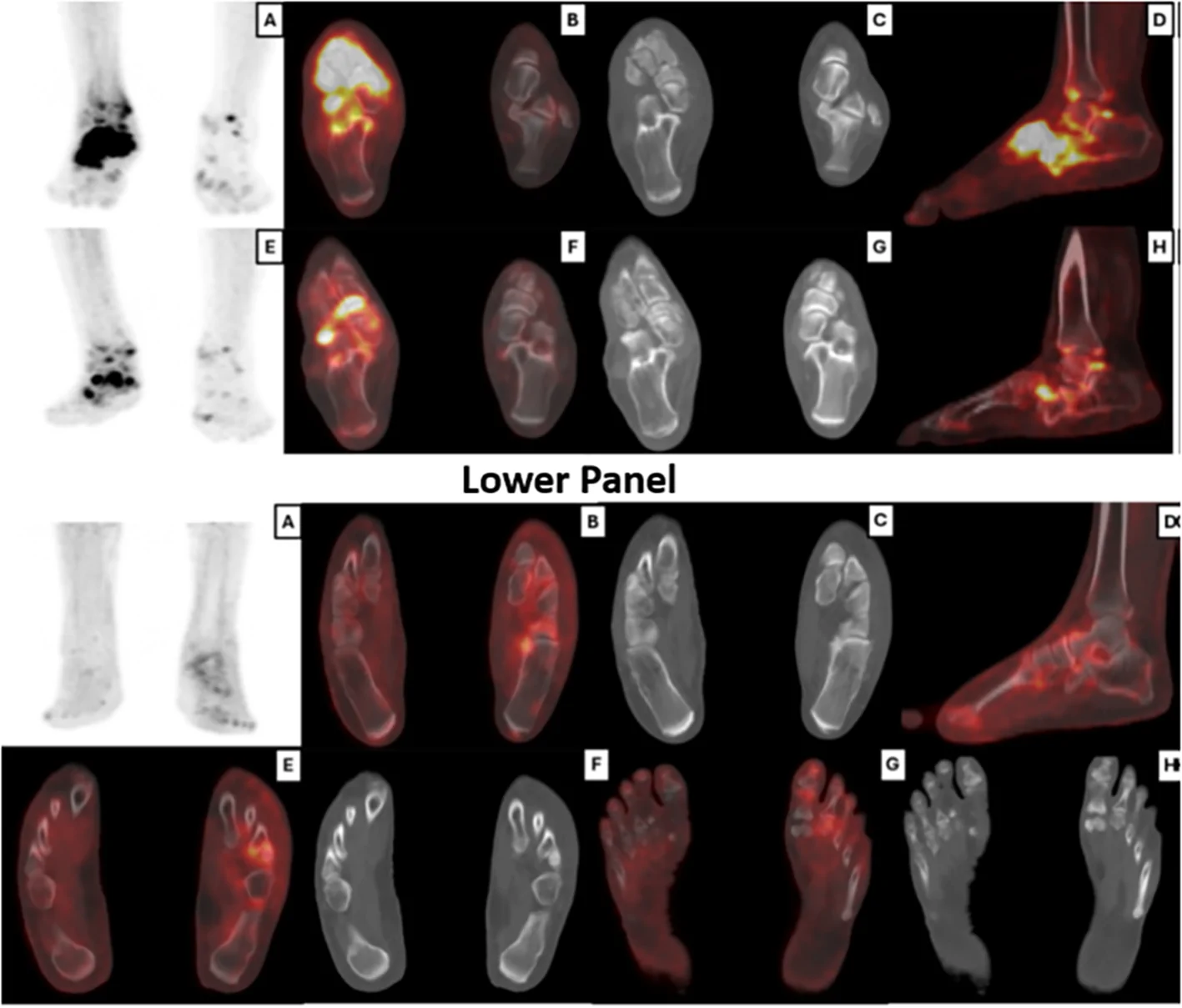
Temporal changes in inter-feet temperature difference and ¹^8^F-NaF PET/CT SUVmax in a patient with sustained remission of active charcot neuro-osteoarthropathy of the foot.

**Table 3:** Exploratory univariate Cox regression analysis of factors associated with recurrence of active CNO.

Parameters	Hazard ratio	95% CI
Age	0.907	0.800-1.029
Duration of Diabetes	0.778	0.604-1.002
Baseline Inter-feet Temperature difference	1.666	0.802-3.460
Calcium	1.193	0.164-8.668
Phosphate	1.300	0.360-4.693
Alkaline Phosphatase	0.990	0.945-1.037
iPTH	0.972	0.912-1.036
25(OH)D	1.025	0.974-1.079
P1NP	0.995	0.970-1.021
βCTX	1.000	0.996-1.003
HbA1c	0.859	0.349-2.113
BMI	0.918	0.725-1.162
Delta SUVmax	0.970	0.941-0.999
SUVmax(R) change (%)	0.904	0.818-0.999

Due to the limited number of recurrence events (n=4), multivariable analysis was not performed. Results represent exploratory univariate Cox proportional hazards regression analyses.

## Discussion

This study characterizes the role of ^18^F-NaF PET-CT in the diagnosis of unilateral active CNO of the foot in people with diabetes, in monitoring bone remodeling activity during treatment, and in exploratory assessment of recurrence risk. We found that a SUVmax of ≥10.3 had high sensitivity for diagnosing active CNO, with specificity dependent on the comparison group. Greater reductions in Delta SUVmax from baseline to clinical remission showed an exploratory association with a lower risk of recurrence.

CNO is associated with a significant morbidity and a three times higher mortality rate than individuals with diabetes with neuropathy (12). It is observed that 95% of referring physicians miss the diagnosis of CNO of the foot at onset (13). The chances of foot deformity due to CNO are significantly increased if the diagnosis is delayed more than four weeks from the onset of symptoms. The active CNO progresses to stage 2 or stage 3 (Eichenholtz classification) before it is diagnosed and the delay in diagnosis of active CNO contributes to the development of foot deformities with rise in inflammatory markers (14). Therefore, a diagnostic modality that can pick up early changes of bony inflammation in patients presenting with warm, swollen feet is the need of the hour to prevent long-term complications of active CNO.

With the limitations of clinical examination to diagnose active CNO of the feet in people with diabetes, various diagnostic modalities have been utilized for the diagnosis of CNO, including X-ray and MRI. Conventional imaging modalities for the diagnosis of active CNO have demonstrated variable diagnostic performance. Plain radiography has a relatively low sensitivity for early disease (15), as osseous changes are often absent during the initial stages (16). In contrast, MRI, the current imaging modality of choice, has reported sensitivities ranging from 77% to 100% in various studies and specificity from 80% to 100% (17–19). Similarly, three-phase bone scintigraphy is highly sensitive (approximately 88%) but lacks specificity because increased tracer uptake may also occur in infection, trauma, and other conditions associated with increased bone turnover (20, 21). Although ^18^F-FDG PET/CT has shown promising diagnostic performance in small studies, its use has largely been confined to differentiating active CNO from osteomyelitis (22) (**Table 4**).

**Table 4:** Comparing the utility of various modalities in active CNO diagnosis, monitoring and assessing recurrence risk.

Study	Imaging modality	Patients	Sensitivity	Specificity	Quantitative biomarker	Role in follow-up/recurrence
Yousaf S. et al. (17)	MRI	Review	77–100%	80–100%	No	Used for follow-up in several studies but no validated quantitative marker for assessing recurrence
Bone scintigraphy studies (20, 21)	Three-phase bone scintigraphy	Various	Around 88%	Low	No	Diagnostic only; not evaluated for recurrence prediction
Basu et al. (22)	¹^8^F-FDG PET/CT	63 patients of four groups	100%	93.8%	SUVmax	Diagnostic differentiation of CNO and osteomyelitis. Not specific for active CNO.
Ruotolo et al. (28)	¹^8^F-FDG PET/CT	25 patients of Stage 0 CNO	—	—	SUVmax	Serial FDG-PET used for monitoring treatment response; not evaluated for recurrence
Previous ¹^8^F-NaF PET/CT case series (9)	¹^8^F-NaF PET/CT	3	Descriptive	—	SUVmax	Demonstrated increased uptake in active CNO; no longitudinal or recurrence assessment
Present study	¹^8^F-NaF PET/CT	33 active CNO	100%	92.3%	SUVmax	First study to evaluate quantitative SUVmax changes as an exploratory biomarker for monitoring remission and predicting recurrence

Active CNO is characterized by increased bone remodeling following activation of the receptor activator of nuclear factor kappa-B ligand (RANKL) and an alteration of the RANKL/osteoprotegerin (OPG) ratio (23). This process is limited to the bones and joints of the feet. The fluoride tracer is incorporated into the bone matrix, providing an indication of bone remodeling. The ¹^8^F-NaF PET-CT scan, primarily used in malignancies with suspected bone metastases, is quite specific for bone-related pathologies and has shown greater sensitivity than fluorodeoxyglucose positron emission tomography (FDG-PET) in detecting such lesions (24).

The two false-positive results in this cohort both occurred in patients with structural bone injury, a stress fracture and foot trauma, while all four patients with purely soft-tissue pathology were correctly classified. This pattern is consistent with the biological basis of the tracer, ^18^F-NaF is incorporated into bone matrix at sites of active osteoblastic remodelling and does not accumulate in soft-tissue inflammation. Reduced specificity against symptomatic mimickers therefore reflects the expected behaviour of a bone-remodelling tracer rather than non-specific uptake, and indicates that ^18^F-NaF PET/CT distinguishes active CNO from soft-tissue mimickers such as cellulitis with greater confidence than from conditions involving concurrent osseous injury. In clinical practice the differentiation of active CNO from fracture rests on the anatomical distribution of uptake on the co-registered CT and on the clinical history, rather than on SUVmax alone. These findings underscore the selective uptake of fluoride in areas of active bone remodeling, as seen in patients with CNO compared to CNO mimickers and healthy individuals. The significant difference in radiotracer uptake in foot bones in patients with CNO emphasizes the diagnostic potential of ^18^F-NaF PET-CT in detecting active CNO. These findings suggest that ^18^F-NaF PET/CT has the potential to complement clinical assessment and MRI in the evaluation of suspected active CNO, although further validation in larger independent cohorts is warranted.

The remission of active CNO is conventionally assessed by periodic infrared thermometry, and clinical remission is defined by an inter-feet temperature difference < 2 °C (3). Temperature monitoring for active CNO has several limitations, including the absence of standardized thresholds of temperature to indicate ongoing bone inflammation in active CNO, variability in the measurement techniques, and susceptibility to external factors such as ambient temperature and physical activity (25). The clinical remission cutoff remains arbitrary, lacks robust supporting data, and there is no quantifiable parameter to predict recurrence after initial clinical remission. In spite of that it is the only definition of clinical remission as per the IWGDF guideline (8) and thereby clinical remission in this study was defined entirely by clinical examination and infrared dermal thermometry, in accordance with current IWGDF guidance, and ^18^F-NaF PET/CT played no part in that determination. The reduction in SUVmax observed at remission should therefore not be interpreted as an alternative definition of remission, but as a quantitative measure of residual bone remodelling at the time clinical remission was declared. The wide range of residual SUVmax values at this timepoint indicates that clinically defined remission encompasses a spectrum of underlying bone activity, which may in part account for the recognised risk of recurrence after apparently successful treatment.

Although recurrence (reactivation) of active CNO has been described in longitudinal cohorts (4), previous studies have primarily identified clinical risk factors such as obesity and non-compliance, or have reported recurrence rates during follow-up (26, 27). Imaging studies have focused on monitoring disease activity using FDG-PET rather than predicting recurrence (28). Semi-quantitative MRI-based approaches are currently being investigated (29, 30), but a robust quantitative marker for monitoring disease activity remains lacking. However, persistent bone marrow edema may be observed despite clinical remission (31), potentially limiting its ability to quantify ongoing disease activity. In contrast, ^18^F-NaF PET-CT directly reflects bone remodeling activity and provides a quantitative measure through SUVmax. Therefore, ^18^F-NaF PET-CT may complement rather than replace MRI in the assessment of active CNO. Similarly, bone scans lack quantitative value and are unable to assess recurrence risk reliably, while FDG-PET uptake is not specific to bone remodeling. To our knowledge, no previous study has evaluated quantitative ¹^8^F-NaF PET/CT parameters, including SUVmax reduction, as an imaging biomarker for recurrence risk after clinical remission.

^18^F-NaF uptake reflects osteoblastic activity and bone remodelling rather than inflammation specific to CNO. Elevated uptake may therefore occur in trauma, fracture, degenerative disease, infection and other conditions associated with increased bone turnover, as observed among the symptomatic non-CNO patients in this cohort. ^18^F-NaF PET/CT is accordingly best positioned as a high-sensitivity quantitative adjunct within a diagnostic pathway in which specificity is contributed by clinical assessment and MRI, rather than as a standalone diagnostic test.

The present study suggests that ¹^8^F-NaF PET-CT provides a quantitative assessment of bone remodeling activity, both at diagnosis and at the time clinical remission. A reduction in SUVmax at the ROI reflects a decrease in bone remodeling activity. Greater reductions in Delta SUVmax and SUVmax(R) change % until clinical remission showed an exploratory association with a lower risk of recurrence. The observed association between Delta SUVmax at remission and subsequent recurrence is temporally coherent, in that the imaging measurement preceded the clinical event in every case. Given the limited number of recurrence events, these findings should be considered hypothesis-generating and require validation in larger prospective cohorts before clinical application. We observed a 13.8% recurrence following initial clinical remission, consistent with the previous reports (12–33%) (4–5). Our findings suggest that ¹^8^F-NaF PET-CT has potential as a quantitative marker of bone remodeling following clinical remission, and that residual activity at remission may warrant further investigation in relation to recurrence risk.

Patients who experienced recurrence had a shorter reported duration of diabetes than those with sustained remission (median 5 vs 15 years), and were also younger. A shorter duration of diabetes would generally be expected to associate with less severe microvascular and neuropathic complications, so the direction of this difference does not obviously favour a higher risk of recurrence. With only four recurrence events, however, adjustment was not feasible and no inference can be drawn either way; the difference may equally reflect chance, and baseline heterogeneity between the subgroups cannot be excluded. However, given the rarity of the disease, such heterogeneity is likely a reflection of clinical practice.

^18^F-NaF PET-CT is a modality that has regional scanning potential specifically for small areas of involvement in foot bones and joints, with a limited radiation exposure. Although the composite clinical and MRI reference standard reflects current guidance, it is not fully independent of imaging. ¹^8^F-NaF PET/CT was not incorporated into the reference standard and readers were mutually blinded, but the absence of an independent gold standard remains inherent to diagnostic studies in active CNO. A major limitation of the study is the small number of recurrence events (n=4), which precluded reliable multivariable modeling. Consequently, the observed associations between SUVmax changes and recurrence should be considered exploratory and hypothesis-generating and require validation in larger prospective cohorts. In addition, the cost may restrict its widespread implementation in routine clinical practice, particularly in resource-constrained settings. Also, our results may not be applicable in cases of bilateral CNO, CNO with PAD, previous foot surgeries or those with recent anti-resorptive therapies.

Imaging was performed on two PET/CT systems and uptake time varied between 45 and 60 minutes, both of which may influence absolute SUV values. All serial scans within a patient were performed on the same scanner, and the longitudinal analyses rely on within-patient percentage change in SUVmax rather than absolute values, which reduces the influence of these sources of variation.

SUVmax measurements were performed by a single reader, and interobserver and intraobserver reproducibility were not formally assessed. A fixed-volume three-dimensional VOI was placed at the site of maximum uptake and referenced to a defined bone on the co-registered CT, which standardizes placement across serial scans, but this does not substitute for a formal reproducibility analysis. The diagnostic threshold was derived and evaluated within the same cohort. Although internal validation indicated only modest optimism, the position of the threshold is determined by a small number of observations near the boundary between groups, and external validation in an independent cohort is required before clinical application. SUVmean and volumetric metabolic parameters were not recorded prospectively and could not be derived retrospectively; their addition would be informative.

Multiple univariate comparisons were performed without adjustment for multiple testing; therefore, the observed associations should be interpreted as exploratory and hypothesis-generating and needs to be tested in large cohorts.

## Conclusion

^18^F-NaF PET/CT provides a quantitative measure of bone remodelling that may assist in the assessment of unilateral active CNO of the foot in people with diabetes presenting with asymmetric foot swelling. In this exploratory single-centre study, greater reductions in SUVmax between baseline and clinical remission showed an association with lower risk of subsequent recurrence. The diagnostic threshold identified here is study-derived and the prognostic findings are based on a small number of events; both require validation in independent prospective cohorts before clinical application.

## Data Availability

The raw data supporting the conclusions of this article will be made available by the authors, without undue reservation.
